# Surface Morphology and Electrochemical Behavior of Microstructured Cu Electrodes in All-Solid-State Sodium Batteries

**DOI:** 10.3390/molecules30173493

**Published:** 2025-08-25

**Authors:** Tomás Prior, Joana Figueira, Ângela Freitas, David Carvalho, Beatriz Moura Gomes, Manuela C. Baptista, Hugo Lebre, Rodrigo Martins, Luís Pereira, Joana Vaz Pinto, M. Helena Braga

**Affiliations:** 1 MatER—Materials for Energy Research Laboratory, Engineering Faculty, University of Porto, 4200-465 Porto, Portugal; up201906963@edu.fc.up.pt (T.P.); up201906619@up.pt (Â.F.); up201805007@up.pt (B.M.G.); up200501276@fe.up.pt (M.C.B.); up201604718@up.pt (H.L.); 2 Centro de Investigação de Materiais—CENIMAT|i3N, Department of Materials Science, School of Science and Technology, NOVA University Lisbon and Centro de Excelência de Microelectrónica e Optoelectrónica de Processos—CEMOP/UNINOVA, Campus de Caparica, 2829-516 Caparica, Portugal; j.figueira@fct.unl.pt (J.F.); dms.carvalho@campus.fct.unl.pt (D.C.); rfpm@fct.unl.pt (R.M.); lmnp@fct.unl.pt (L.P.); jdvp@fct.unl.pt (J.V.P.); 3 LAETA—Associated Laboratory of Energy, Transports and Aeronautics, 4200-465 Porto, Portugal

**Keywords:** all-solid-state batteries, sodium-ion batteries, microstructured current collectors, copper thin films, colloidal lithography, electrochemical impedance spectroscopy, atomic force microscopy, interface engineering

## Abstract

The integration of microstructured current collectors offers a potential pathway to enhance interface properties in solid-state battery architectures. In this work, we investigate the influence of surface morphology on the electrochemical performance of Zn/Na_2.99_Ba_0.005_OCl/Cu electrodeless pouch cells by fabricating copper thin films on microstructured parylene-C substrates using a combination of colloidal lithography and reactive ion etching. O_2_ plasma etching times ranging from 0 to 15 min were used to tune the surface topography, resulting in a systematic increase in root-mean-square roughness and a surface area enhancement of up to ~30% for the longest etching duration, measured via AFM. Kelvin probe force microscopy-analyzed surface potential showed maximum differences of 270 mV between non-etched and 12-minute-etched Cu collectors. The results revealed that the chemical potential is the property that relates the surface of the Cu current collector/electrode with the cell’s ionic transport performance, including the bulk ionic conductivity, while four-point sheet resistance measurements confirmed that the copper layers’ resistivity maintained values close to those of bulk copper (1.96–4.5 µΩ.cm), which are in agreement with electronic mobilities (−6 and −18 cm^2^V^−1^s^−1^). Conversely, the charge carrier concentrations (−1.6 to −2.6 × 10^23^ cm^−3^) are indirectly correlated with the performance of the cell, with the samples with lower CCC_bulk_ (fewer free electrons) performing better and showing higher maximum discharge currents, interfacial capacitance, and first-cycle discharge plateau voltage and capacity. The data were further consolidated with Scanning Electron Microscopy and X-ray Photoelectron Spectroscopy analyses. These results highlight that the correlation between the surface morphology and the cell is not straightforward, with the microstructured current collectors’ surface chemical potential and the charge carriers’ concentration being determinant in the performance of all-solid-state electrodeless sodium battery systems.

## 1. Introduction

The European Union’s goal of achieving net-zero carbon emissions necessitates a fundamental rethinking of the energy systems that underpin modern society. Central to this transformation is the ongoing electrification of various sectors, a trend that continues to accelerate with no indication of fading [1,2]. Despite this progress, significant challenges remain before a complete transition to electrified, fossil fuel-independent systems can be realized across all industries [3,4]. One critical challenge lies in the continued dependence on the centralized electrical grid. While this infrastructure represents a major achievement of modern engineering, it suffers from key limitations. These include difficulties in integrating variable renewable energy sources, vulnerability to extreme weather events, and relatively low transport efficiency over long distances [5]. As a result, decentralized energy solutions, such as on-site energy capture and storage, are gaining traction. These systems are becoming increasingly cost-competitive, offering performance levels that approach or match the cost of grid-purchased energy [6].

Despite considerable advancements in energy storage technologies, conventional battery systems still present important limitations. In electric vehicles, for instance, the weight of current batteries necessitates the use of lightweight structural materials, which can increase overall costs [7,8]. Similarly, on-site storage solutions often require substantial space, a constraint in many residential, industrial, or mobile settings. Looking forward, space exploration is expected to rely heavily on renewable energy systems, further increasing the demand for lightweight, high-energy-density storage technologies [9,10].

Solid-state batteries have emerged as a promising solution to address many of these limitations. They offer several advantages over conventional liquid electrolyte systems, including faster charging rates, enhanced safety due to the absence of flammable components, resistance to dendrite formation, reduced risk of thermal runaway and oxygen release, and broader thermal operating ranges [11,12,13,14]. Furthermore, the inherently higher density of solid-state electrolytes makes them well suited for applications where both weight and volume are at a premium, such as electric vehicles and aerospace technologies [15,16,17,18].

Although lithium-based solid-state batteries have demonstrated high performance, they come with notable drawbacks, particularly in terms of safety and cost. Lithium is less abundant and more difficult to extract compared to other elements, contributing to these concerns [19,20]. In contrast, sodium-based solid-state batteries offer a compelling alternative. Sodium is more abundant, easier to extract, and significantly cheaper, enhancing both the economic and environmental sustainability of these systems [21,22]. However, the larger ionic radius of Na^+^ compared to Li^+^ results in a lower energy density, which could limit their use in applications where weight or spatial constraints are critical.

To overcome this limitation, a promising strategy is the integration of energy storage directly into structural components. Embedding batteries into the load-bearing elements of vehicles, buildings, or spacecraft could reduce overall system weight and free up space, thereby enabling electrification in applications where conventional batteries fall short [23,24,25]. Coaxial structural batteries employing sodium-based solid-state electrolytes [26] aim to address this need by combining the safety and cost advantages of sodium with structural load-bearing functionality. These multifunctional systems have the potential to revolutionize energy storage by seamlessly integrating power supply into the very fabric of vehicles and infrastructure, particularly in weight-sensitive or space-constrained environments such as electric transportation and space exploration [27]. The batteries developed by Braga’s group utilize a sodium-based solid-state electrolyte, specifically Na_2.99_Ba_0.005_OCl. This material exhibits high ionic conductivity (10^−2^ S cm^−1^) and a high pyroelectric coefficient (5.37 C.m^−2^.°C^−1^), due to its ferroionic behavior [28,29]. In these systems, the electrolyte is employed in a configuration that does not include a traditional anode or cathode. The current collectors, such as zinc or aluminum for the anode side and carbon or copper for the cathode side, create a chemical potential difference. This drives the plating and stripping of sodium ions during the charge and discharge processes [30]. The efficiency of ion plating onto the surface of the current collectors is a critical factor in the performance of anode-less batteries, particularly when no active electrode materials are present [31]. In such cases, the total capacity of the battery relies entirely on the reversible deposition of sodium ions [32].

One strategy to enhance battery performance, whether active materials are used or not, is to alter the topography of the current collectors. Microstructured electrodes are widely studied in supercapacitor research due to their ability to significantly increase surface area, which, in turn, boosts double-layer capacitance [33,34]. In the context of batteries, surface modification of current collectors offers additional benefits beyond increased surface area. Three-dimensional microstructured architectures can improve the mechanical stability of the electrode–electrolyte interface, enhance adhesion, and suppress dendrite formation, thereby contributing to both safety and long-term cycling stability [35,36,37,38,39,40]. Pseudocapacitors, which represent an intermediate class between batteries and supercapacitors [41], also benefit from the use of microstructured current collectors. Since these systems rely heavily on surface-confined redox reactions, the geometry and morphology of the current collector play a crucial role in their electrochemical performance [42,43].

In coaxial structural batteries, the biggest radius current collector must also be flexible. This is usually achieved through the malleability of copper, aluminum, or zinc sheets, or the use of flexible substrates like poly(ethylene naphthalate) (PEN)/poly(ethylene terephthalate) (PET) in the case of thin-film deposition [44].

To this end, poly(p-xylylene) derivatives, commonly known as parylenes, have been widely explored by the scientific community for several applications, either as passive coatings or active device components. Poly(chloro-para-xylylene), or parylene-C, belongs to this class and stands out as a flexible and transparent dielectric polymer, being widely used due to its chemical inertness and conformability properties [45,46].

The tailoring of the parylene surface roughness, increasing its surface area, can be obtained through a colloidal lithography process. This method begins with the deposition of a monolayer of self-assembled colloidal polystyrene microspheres through the Langmuir–Blodgett technique. The obtained close-packed honeycomb structure is used as a physical mask for the subsequent surface patterning through reactive ion etching under O_2_ plasma (RIE). Both parylene and polystyrene spheres are etched under O_2_ plasma, allowing the precise micro-patterning of parylene with tapered microstructured features, replicating the same periodicity as the physical etching mask used [47,48]. The modification of surface topography via this method is particularly attractive for industrial applications owing to its low (ambient) processing temperature and scalability [47].

In this work, electron-beam evaporation was used to deposit a copper thin film onto various microstructured parylene surfaces prepared with different oxygen RIE durations. These samples were then characterized and employed as positive current collectors in Zn/Na_2.99_Ba_0.005_OCl/Cu electrodeless pouch cells. The goal of this study is to investigate the effect of microstructured copper current collectors on solid-state battery performance, focusing on how the surface morphology influences the electrochemical behavior of the system. The use of pouch cells, instead of structural batteries, enables higher reproducibility and reduced material usage while maintaining a similar electrochemical configuration to that of coaxial structural batteries.

## 2. Results and Discussion

This section is organized into two subsections: The first presents the results obtained from the characterization of the microstructured copper current collectors. The second focuses on the electrochemical performance of pouch cells incorporating these microstructured current collectors. Given the extensive range of characterizations conducted, the data have been consolidated into a series of graphs that summarize key parameters relevant to the analysis.

### 2.1. Characterization of the Microstructured Current Collectors

The characterization of the copper current collectors is an important step in understanding their effect on battery behavior. Topographic features change the nature of electrochemical interfaces between the current collector and the electrolyte. Micro–nanoscale structures were identified and quantified with the use of Atomic Force Microscopy (AFM), while Scanning Electron Microscopy (SEM) and Energy Dispersive X-ray Spectroscopy (EDS) were used to assess the presence of surface defects resulting from the fabrication process. Kelvin Probe Force Microscopy (KPFM) complements the topographic analysis by measuring surface potential, which again can affect interfacial phenomena. X-ray Photoelectron Spectroscopy (XPS) substantiates the KPFM analysis with compositional surface analysis of the samples. Finally, the sheet resistance of the samples was analyzed using the van der Pauw (VdP) method [49], while charge carrier concentration and mobility were determined by Hall-effect measurements [50].

#### 2.1.1. Topographical Characterization

**Figure 1:**
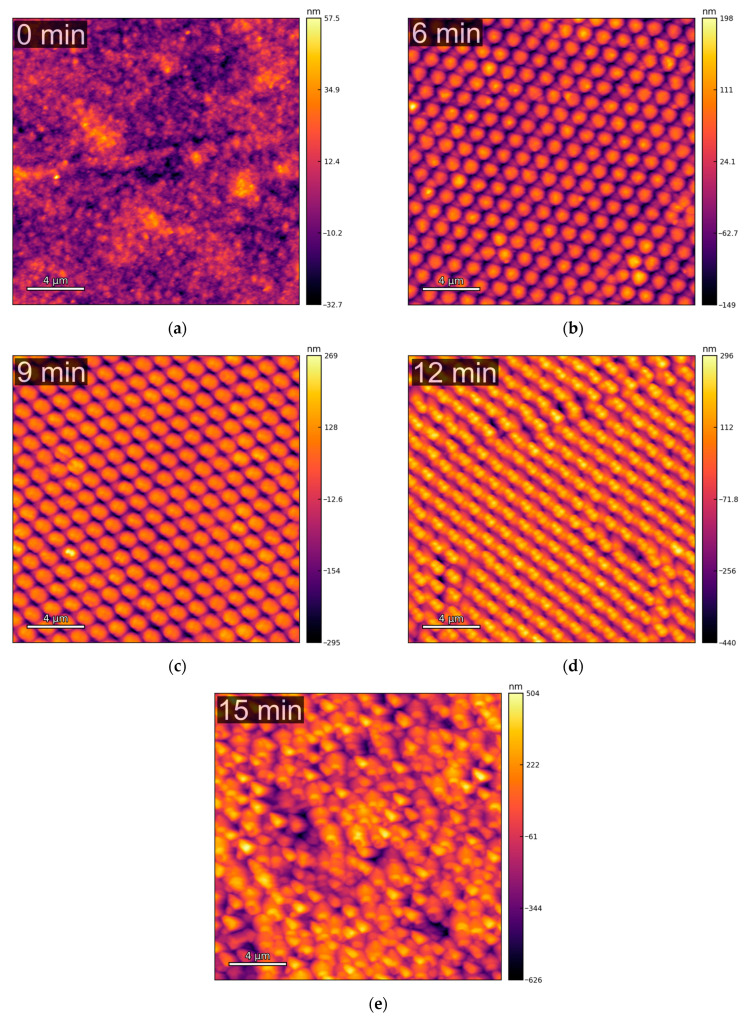
Examples of topography images, obtained via AFM, of the copper current collectors with different etching times: (**a**) 0 min; (**b**) 6 min; (**c**) 9 min; (**d**) 12 min; (**e**) 15 min. The images shown were upscaled to 1024 × 1024 pixels, from the original 256 × 256, for enhanced visual clarity.

The topographical AFM characterization of the copper samples was performed in multiple randomly chosen spots for each sample. Figure 1 shows that the patterning process creates a honeycomb-like pattern of cone-shaped “particles”. The well-structured distribution of the particles, observed for current collectors with 6 (Figure 1b) and 9 min (Figure 1c) of etching time, is gradually lost at higher etching times (Figure 1d,e). This trend is expected, as etching durations beyond 9–12 min can lead to the complete removal of the PS spheres, which serve as the mask for the parylene-C etching. Once the mask is gone, the subsequent etching becomes more irregular and uncontrolled.

**Figure 2:**
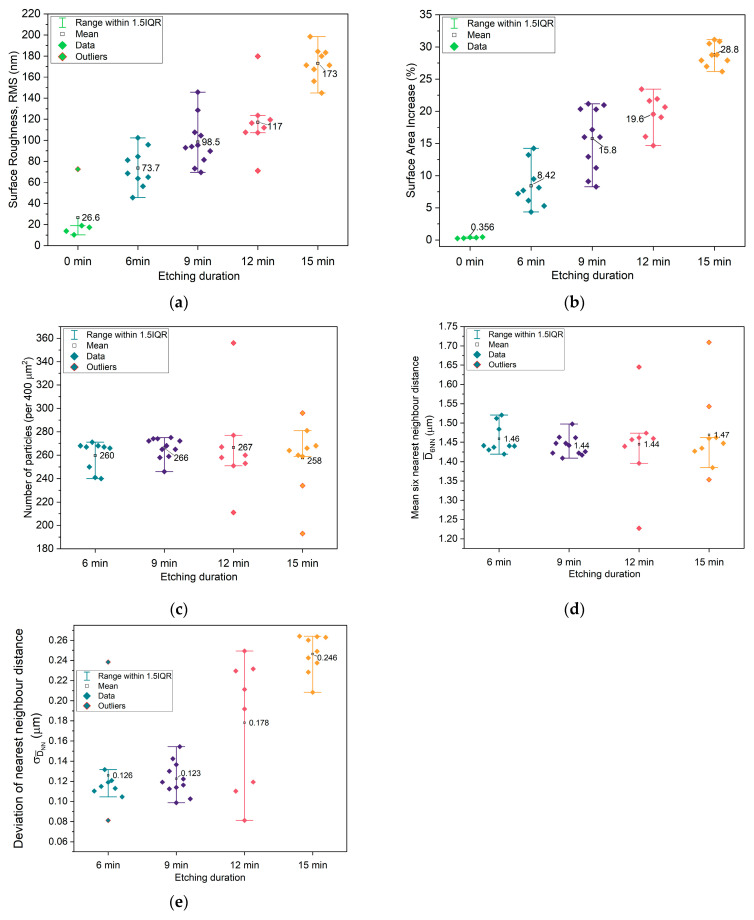
Extracted parameters from topography images: (**a**) root-mean-square (RMS) of topography; (**b**) surface area increase from flat topography (400 μm^2^); (**c**) number of particles; (**d**) average distance between 6 nearest neighbors; (**e**) standard deviation of average nearest-neighbor distance.

To quantitatively evaluate the impact of plasma etching time on the surface morphology of the microstructured copper current collectors, five key topographical parameters were extracted from the AFM images: (1) RMS roughness, (2) relative surface area increase, (3) number of particles, (4) average six-nearest-neighbors distance (D_6NN_), and (5) standard deviation of nearest-neighbor distances (σ_DNN_). These parameters were derived from multiple AFM images for each etching condition, and the resulting distributions were analyzed using box plots to capture both inter-sample trends (e.g., how the mean value evolves with etching time) and intra-sample variability (e.g., interquartile range and the presence of outliers) (Figure 2).

**RMS Roughness:** A systematic increase in RMS roughness was observed with longer etching times, confirming that extended O_2_ plasma exposure enhances the surface relief. This behavior is consistent with the expected deepening of surface features as more parylene-C-masked material is selectively removed. Notably, the interquartile range (IQR) narrows at higher etching durations, suggesting that the roughness not only grows but also becomes more spatially homogeneous across the surface (Figure 2a).

**Surface Area Increase:** The evolution of surface area follows a similar trend, increasing steadily with etching time in tandem with RMS roughness, up to an average increase of 28.8 ± 1.17% for the 15 min sample. This is expected, as the development of deeper or taller topographical features increases the actual surface area relative to the projected 2D footprint. The reduced IQR at higher etching times also points toward a more uniform topographic landscape, which may enhance the consistency of the electrochemical interface (Figure 2b).

**Number of Particles:** Across most etching times, the average number of detectable topographic features remains relatively constant, indicating that the lateral pattern fidelity established by Langmuir–Blodgett deposition of polystyrene (PS) spheres is preserved. This suggests that O_2_ plasma etching modifies vertical dimensions without substantially affecting the in-plane distribution of features—at least up to 12 min. At 15 min, however, the appearance of a small number of outliers suggests the onset of local defects or dislocations in the particle lattice, pointing to a possible etching threshold beyond which structural integrity begins to degrade (Figure 2c).

**Average Six-Nearest-Neighbor Distance (D_6NN_):** The average D_6NN_ remains nearly constant (~1.44–1.47 μm) across all etching durations, indicating that the in-plane periodicity governed by the initial PS sphere array is maintained throughout the etching process. This implies that the etching procedure does not significantly perturb the global spatial arrangement of the features. However, the presence of more outliers at extended etching times may reflect localized pattern distortions resulting from the long etching process (Figure 2d).

**Standard Deviation of Nearest-Neighbor Distance (σ_DNN_):** In contrast to D_6NN_, σ_DNN_ exhibits a clear increasing trend with etching time. This indicates growing variability in local feature spacing, which suggests a gradual degradation of short-range order in the feature array. While the global lattice symmetry remains intact, local periodicity becomes less regular, potentially influencing surface potential uniformity and leading to uneven ion nucleation during subsequent sodium plating. This degradation in spatial order complements the observed changes in particle count and D_6NN_, emphasizing that although the layout is nominally preserved, longer etching introduces disorder at the micro–nanoscale (Figure 2e). It is noteworthy highlight that longer etching in principle corresponds to lower *information* and, therefore, increased entropy. However, Figure 2e shows that the sample with lowest average σ_DNN_ is the one etched for 9 min, while the sample showing the lowest average σ_DNN_ values is the one etched for 12 min, which will later correspond to the current collectors/electrodes of the best-performing cells.

**Figure 3:**
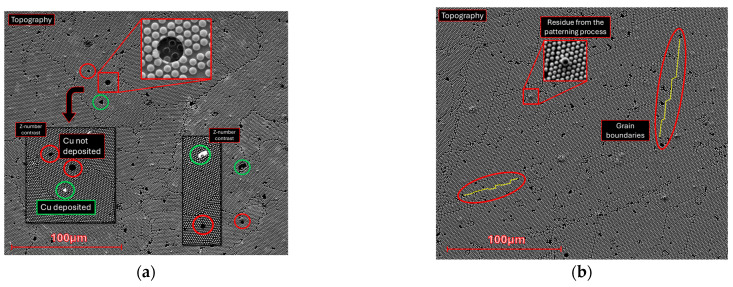
SEM images of the microstructured copper surfaces. Topographic features such as defects, residue from the patterning process, and grain boundaries are highlighted in red, green, and yellow, respectively. Samples’ etching: (**a**) 6 min; (**b**) 9 min.

Microscale SEM images (Figure A6) highlight some features that are less perceptible by AFM. Defects such as unmasked areas and particle grain boundaries—although common in all samples, as highlighted in Figure 3—comprise a small part of the total area. Atomic number contrast images (Figure A6, right-hand column) and EDS (Figure A7) show that, in most cases, copper was still deposited in those areas.

**Figure 4:**
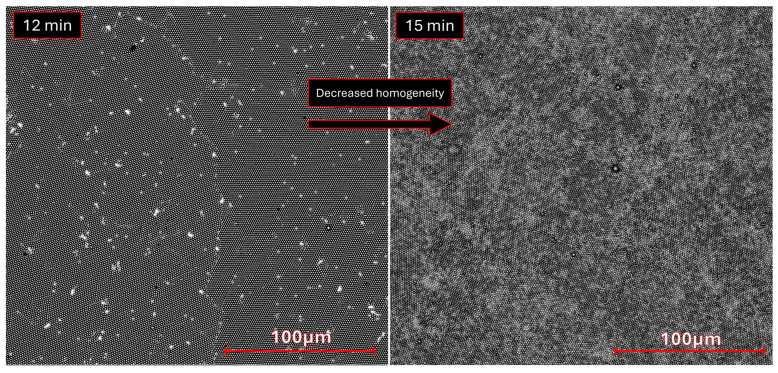
Atomic number contrast SEM images, obtained with backscattered electrons, for the microstructured copper samples etched for 12 min (**left**) and 15 min (**right**), highlighting the increased inhomogeneity resulting from the prolonged etching process with particles forming bridges, which result in the formation of two phases with not too dissimilar density, likely Cu^0^/Cu^+^/Cu^2+^ based.

Additionally, SEM images of the sample etched for 15 min (Figure 4) show a less homogeneous surface because of the long etching process. This effect contributes to the increased surface area by creating deeper topographic features besides the cone-like structures highlighted in AFM. Some “bridges” are observed connecting the cones in Figure A6i. Where these bridges are more frequent, a brighter phase is found, likely meaning that charge carriers are exchanged and confined in denser, brighter areas (Figure 4—right). Finally, the obtained wide XPS spectra (Appendix A.3, Figure A8) shows small Cl 2s peaks (from the parylene-C substrate) and no Ti 2p peaks, indicating that copper was deposited across the entire surface without significant defects or residue.

#### 2.1.2. KPFM Characterization

The surface potential ϕ of a sample is characterized using Kelvin Probe Force Microscopy (KPFM). The electrochemical potential of the probe aligns with the electrochemical potential of the material $\bar{\mu }\;=\;\mu +ze\phi$, where μ is the chemical potential of the material or species, z is the valency of the mobile ion, and e is the elementary charge. It depends on the sum of eϕ and the chemical potential μ of the material or species, if z = 1. The work function of a material is WF = $-\bar{\mu }$, accounting for both the chemical potential and the surface potential; in other words, it accounts for bulk and surface phenomena. If the material is electrically insulated and shows no ϕ, then WF = −μ.

The work function of the current collector is a relevant parameter, as it influences the performance of the cell, including the solid-state electrolyte’s ionic conductivity, charge transfer, and deposition behavior [51]. It is also an indicator of the level of oxidation on the copper surface.

**Figure 5:**
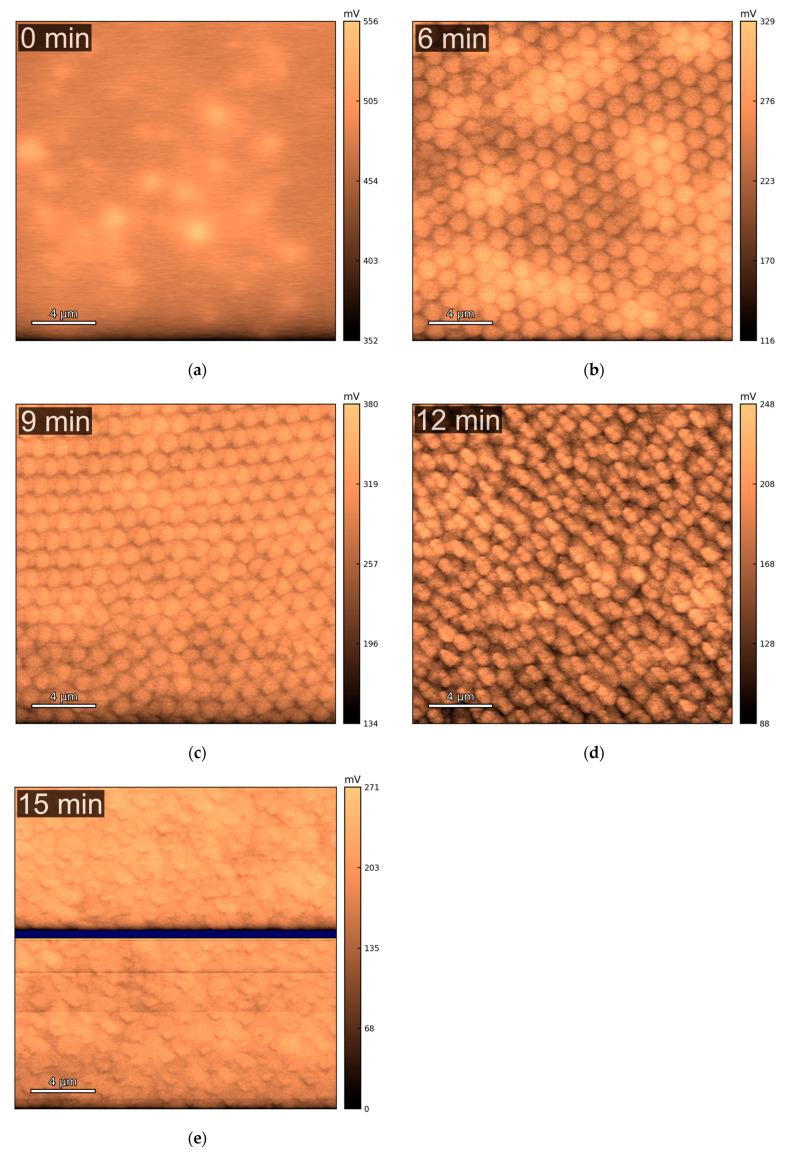
Maps of surface potentials $\phi_{Cu}$, where ${\bar{\mu }}_{Cu}-{\bar{\mu }}_{tip}\;=\;0\;=\;\mu_{Cu}-\mu_{tip}\;=\;\;e(\phi_{tip}-\phi_{Cu})$, where the tip is calibrated to be equal to the standard hydrogen electrode $\mu_{tip}\;=\;0\;eV\;vs.\;SHE\;=\;-4.44\;\mathrm{eV}\;\mathrm{vs}.\;\mathrm{electrons}\;\mathrm{at}\;\mathrm{rest}\;\mathrm{in}\;\mathrm{vaccum}$, obtained via KPFM, of the copper current collectors with different etching times: (**a**) 0 min; (**b**) 6 min; (**c**) 9 min; (**d**) 12 min; (**e**) 15 min. Areas masked in dark blue represent excluded points where the surface potential dropped below 0 V due to charge accumulation. These areas were not considered for obtaining the average surface potential, as the focus is herein given to the background.

**Figure 6:**
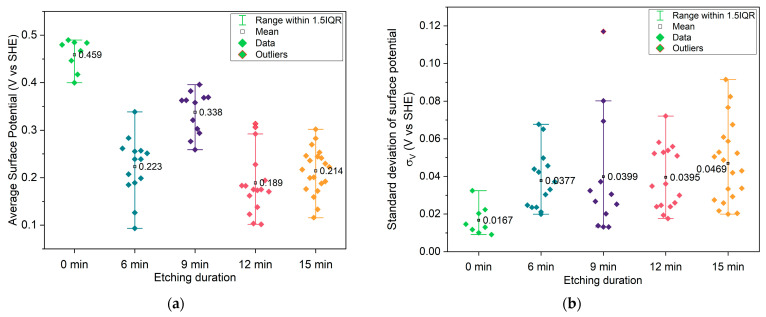
(**a**) Average surface potential of each KPFM image for different etching durations; (**b**) standard deviation of surface potential of each KPFM image for different etching durations.

**Figure 7:**
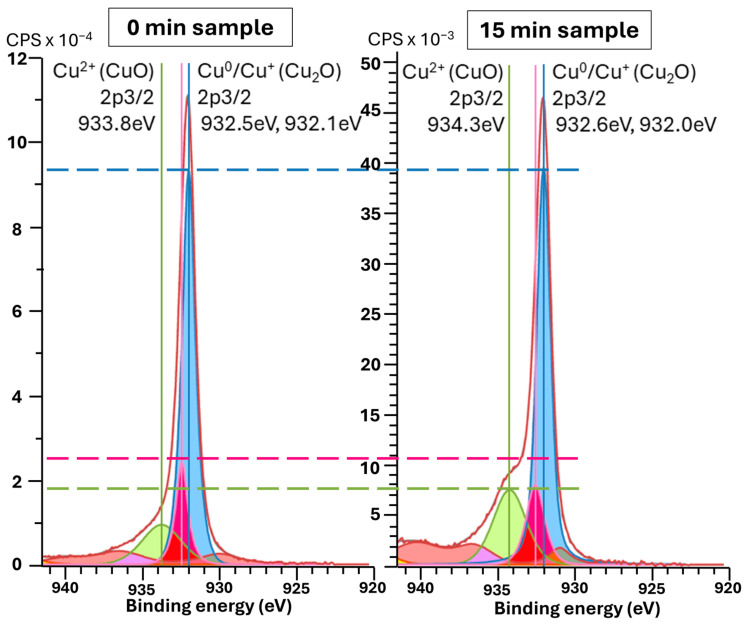
Comparison of XPS spectra, near Cu 2p_3/2_ peaks, obtained for the microstructured copper samples etched for 0 (**left**) and 15 (**right**) minutes. Cu^+^ peaks colored in blue, Cu^0^ peaks colored in rose, Cu^2+^ peaks colored in green and loss features colored in red.

Figure 5 and Figure 6a show the average surface potential for the different copper samples, all measured relative to the tungsten WF ≈ 4.44 eV (μ = −4.44 eV) of the calibrated tip, corresponding to 0 V vs. SHE (standard hydrogen electrode). The 0 min sample exhibits a surface potential in the range of +0.45 V vs. SHE, which aligns with the expected values for clean, metallic copper surfaces (typically +0.34 V Cu^2+^/Cu(s) vs. SHE and +0.52 V Cu^+^/Cu(s) vs. SHE) [52], suggesting minimal surface oxidation. In contrast, the microstructured samples show systematically lower average surface potentials, indicating a decrease in work function relative to the flat vacuum reference. Two competing effects may explain this trend: First, the cone-like surface topography introduced by etching can lead to local charge redistribution. Electrostatic field concentration at the sharper valleys may promote electron accumulation, thereby decreasing the work function, while increasing the local surface potential. The planar top regions between these valleys may exhibit lower electron density, effectively increasing the local work function. This effect is directly observed in the raw KPFM images, where the tips of the cones show higher surface potential vs. SHE than the surrounding valleys. In structured samples, this would lead to lower average surface potential compared to the unstructured sample. This is also noticeable in Figure 6b, with structured samples having higher deviation in surface potential. A second important effect is oxidation, which becomes more significant for microstructured surfaces due to their increased surface area. The formation of CuO (with Cu^2+^) would decrease the work function of the material, thereby lowering the measured surface potential vs. SHE. This oxidation effect likely contributes to the reduced potential observed even at the cone tips, which do not reach the same values as the unstructured, presumably cleaner, flat sample. This idea is substantiated by the XPS Cu 2p ROI spectra (Appendix A.3, Figure A9, left column), which show intense CuO peaks for the 0 and 15 min samples, as shown in Figure 7. Still, in all samples, the Cu LMM Auger ROI (Appendix A.3, Figure A9, right column) show a strong Cu_2_O peak, indicating general surface oxidation. The 6 min samples show a higher relative amount of Cu^0^, and the 15 min samples show almost similar relative distribution of Cu^2+^/Cu^+^/Cu^0^. Native oxidation layers are typically in the order of a few nanometers, which is around the depth of measurement of XPS, while KPFM can be influenced by charge accumulation from the unoxidized underlayer. Thus, XPS only measures the native oxidation layer, while KPFM is influenced by the bulk. This effect is more pronounced on the flat 0 min sample, given that topological effects are reduced. The latter is also corroborated by the surface chemical potential of the 0 min sample being the highest (0.45 V, SHE), corresponding to more Cu^+^/Cu^0^ (0.52 V, SHE) than Cu^2+^/Cu^0^ (0.34 V, SHE), more pronounced than in the other samples showing lowest chemical potentials vs. SHE.

#### 2.1.3. Charge Transport Measurements

**Figure 8:**
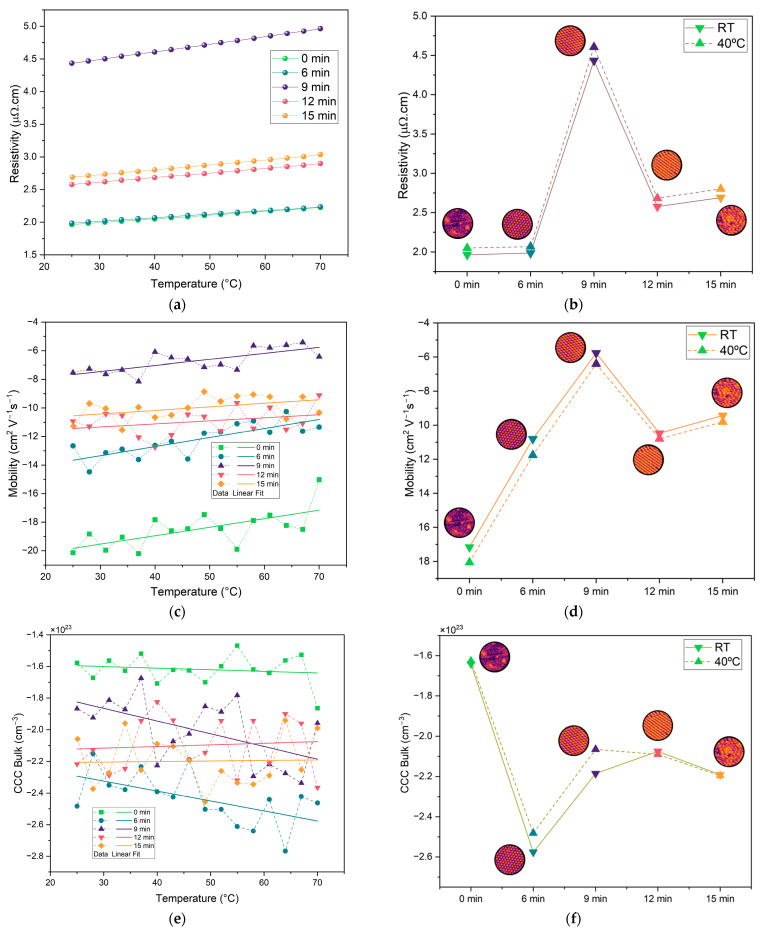
Electrical properties of copper current collectors for each etching duration (0, 6, 9, 12, and 15 min): (**a**) temperature-dependent resistivity measurements for each etching duration; (**b**) comparison between the resistivity at room temperature (RT) and 40 °C for each etching duration; (**c**) temperature-dependent charge carrier mobility for each etching duration; (**d**) comparison between the mobility at RT and 40 °C for each etching duration; (**e**) temperature dependence of calculated bulk charge carrier concentration (CCC_bulk_) across etching durations; (**f**) CCC_bulk_ at RT and 40 °C for each etching duration. Solid and dashed lines in (**b**), (**d**), and (**f**) represent data at RT and 40 °C, respectively. Note: negative values of the mobility and CCC_bulk_ correspond to negative charges, which are electrons in this case.

Given that the current collectors were fabricated by copper deposition, it is important to assess whether this process, along with microstructuring, affects the resistance of the samples and, consequently, the internal resistance of the battery. To investigate this, we performed four-point probe sheet resistance and Hall-effect measurements, as shown in Figure 8. The results in Figure 8a,b display the expected trend of increasing resistivity with temperature, which is typical of metallic conductors. The values of the correspondent resistivity at 40 °C fall within the range reported in the literature [53,54], generally between 2 and 4.5 µΩ.cm. These findings suggest that using copper as a thin film does not significantly increase the resistivity of the current collectors, especially when compared to bulk copper, which has a resistivity of approximately 1.68 µΩ.cm [52]. At 25 °C, the 0 min and 6 min samples showed similar resistivity values of approximately 1.96 µΩ.cm, increasing slightly to approximately 2.06 µΩ.cm at 40 °C. The 12 and 15 min samples also exhibited similar behavior, with resistivity values of 2.57 µΩ cm and 2.69 µΩ cm at 25 °C, and 2.68 µΩ.cm and 2.80 µΩ.cm at 40 °C, respectively. The 9 min sample stood out with the highest resistivity, measuring 4.44 µΩ.cm at 25 °C and 4.60 µΩ.cm at 40 °C. Overall, these results indicate that microstructuring shows no optimal collector based on the resistivity of the copper layers. Conversely, it shows that the 9 min sample is the worst in terms of resistivity.

Hall-effect measurements show electron mobility between 6 and 18 cm^2^.V^−1^.s^−1^, which is lower than typical values for copper (~43 cm^2^.V^−1^.s^−1^) [55], with the highest value being for the non-structured sample. This indicates that the microstructuring process influenced electron mobility. Surface roughness exceeding the 250 nm film thickness introduces local thickness variations, creating an inhomogeneous current path. Nevertheless, even the unstructured reference sample displays a mobility below the bulk value, in agreement with earlier reports that thermally evaporated copper films develop a high density of grain boundaries, which enhance carrier scattering and, thus, lower mobility [56]. The grain boundaries for the microstructured samples are well observed herein in the SEM images (Figure 3). The charge carrier concentration (CCC_Bulk_) is approximately 10^23^ cm^−3^, close to typical values for copper [55]. Overall, the microstructuring has had some impact on the electrical properties of the samples, with structured samples having higher charge carrier concentrations and lower electron mobility, as compared to the non-structured sample.

### 2.2. Pouch Cell Electrochemical Characterization

A total of 15 pouch cells were manually assembled, consisting of three cells for each Cu current collector type, in the configuration Zn/Na_2.99_Ba_0.005_OCl/Cu (Section 3.3 and Section 3.4). The cells were identified by the copper current collector etching duration (0, 6, 9, 12, and 15 min) and by batch number (three batches in total). After assembly, all pouch cells showed an open-circuit voltage (OCV) of approximately 1.1 V. Electrochemical characterization consisted of a sequence of pre-CV PEIS, CV, and post-CV PEIS (Section 3.3 and Section 3.4). The cell corresponding to the 15 min etching duration, batch 2, degraded after the first PEIS, and its data were excluded from the analysis. All cells were tested first at RT and then at 40 °C. RT was not controlled, but it was generally around 25 °C for the days in question. Charge/discharge cycles were performed at RT after the batteries has undergone a long period of rest (>30 days) (Section 3.3 and Section 3.4). The Galvanostatic Intermittent Titration Technique (GITT) was performed at RT after more than 30 days of the charge/discharge cycles.

#### 2.2.1. Potentiostatic Electrochemical Impedance Spectroscopy

**Figure 9:**
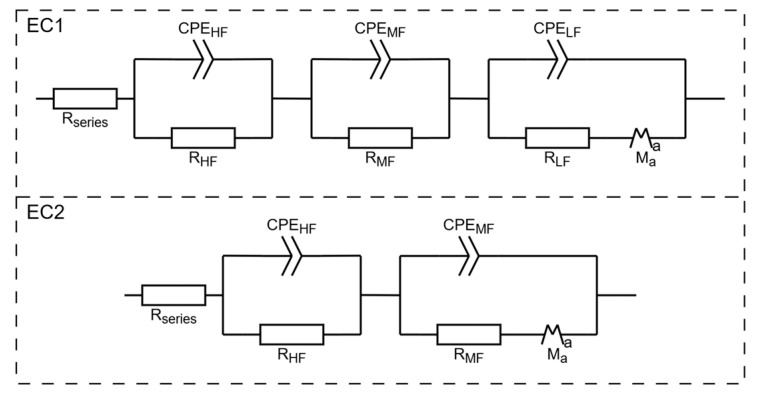
Two equivalent circuits used for modeling the PEIS. CPE: Constant Phase Element, Ma: modified restricted diffusion element. Suffixes: HF (high frequency), MF (mid-frequency), LF (low frequency).

The results obtained from PEIS were analyzed using equivalent circuit fitting (EC fit). Two distinct equivalent circuits, as shown in Figure 9, were used in the analysis. The circuit EC2 was preferentially used. Circuit EC1 was used if a third, low-frequency semicircle was present. This analysis allowed for the extraction of key parameters, including the resistance in series (R_series_), high-frequency capacitance (C_HF_), and resistance (R_HF_) corresponding to the left-most semicircle, as well as the mid-frequency capacitance (C_MF_) and resistance (R_MF_) corresponding to the intermediate semicircle. Pseudo-capacitances (C_HF;MF;LF_) were calculated from the respective CPE and R elements via Equation (1), where Q is the resulting capacitance of the CPE:

C = Q^(1/α)^ ∙ R^(1−α)/α^ (1)

The extracted parameters for each cell are displayed in Appendix A (Figure A2, Figure A3 and Figure A4). From these parameters, the mean values were calculated for comparison across etching durations.

**Figure 10:**
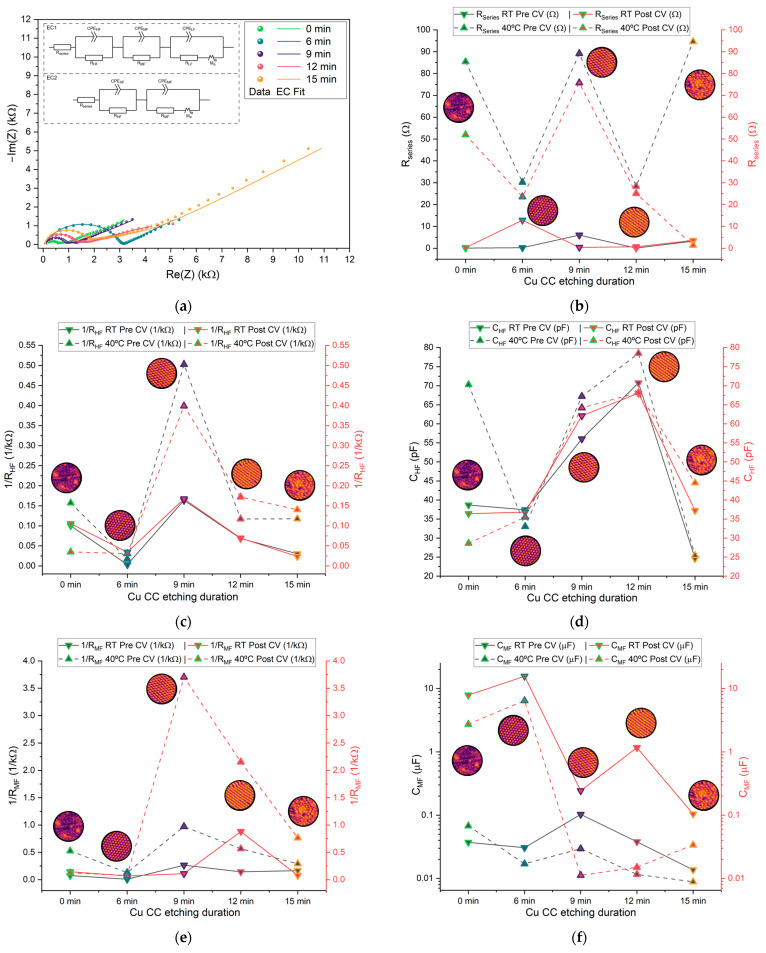

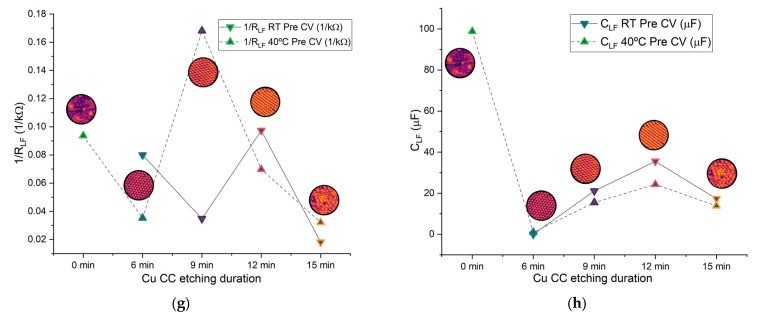
(**a**) Plots of selected PEIS cycles for different Zn/Na_2.99_Ba_0.005_OCl/microstructured Cu cells; (**b**–**f**) average values of different PEIS parameters for cells with each current collector type at RT and 40 °C, pre- and post-CV; (**b**) resistance in series $R_{series}$; (**c**) 1/$R_{HF}$; (**d**) $C_{HF}$; (**e**) 1/$R_{MF}$; (**f**) $C_{MF}$; (**g**) 1/$R_{LF}$; (**h**) $C_{LF}$. The low-frequency semicircle was not present on 0 min cells at RT. Most cells did not show a post-CV LF semicircle; therefore, this data was excluded from the comparison.

The resistance in series $R_{series}$ (Figure 10a) is influenced by multiple factors, including the electrolyte, electrode materials, and contact resistance. Given the microstructured thin-film nature of the current collectors used in this study, $R_{series}$ is a particularly relevant parameter for comparison. The samples etched for 0 min, which feature a flat surface, exhibit $R_{series}$ values comparable to those of the microstructured samples, indicating that the structuring process has minimal impact on this parameter. The pouch cells etched for 9 min display a slightly higher average $R_{series}$, which aligns with the elevated resistivity observed using the vdP method (Figure 8b). The values shown in Figure 10b are consistent with the results previous studies employing bulk copper sheet electrodes [31], indicating that the use of thin films does not significantly alter $R_{series}$.

The high-frequency (HF) semicircle is primarily associated with electrolyte bulk phenomena, although grain boundary effects may also influence this region [57,58]. C_HF_, the ideal capacitor of thickness *d* and surface area *A*, in principle is not affected by the electrodes’ surface except by the bias created by the difference in chemical potential, which is responsible for the driving force—the electric field that pulls the cations towards the interfaces while cycling. These bulk cations Na^+^ face a resistance,

(2) $$R_{HF}\;=\;\rho \frac{d}{A}$$

where ρ is the ionic resistivity 1/ρ = σ, and σ is the ionic conductivity of the electrolyte.

The corresponding capacitance $C_{HF}$ and resistance $R_{HF}$ values from the HF RC equivalent circuits are presented in Figure 10c,d. The capacitances at HF range from 0.01 to 0.1 nF, which are typical for bulk processes in solid-state devices; ${1/R}_{HF}$ is in the order of 10^−4^ Ω^−1^ and exhibits an optimal value 0.5 × 10^−3^ Ω^−1^. Notably, the samples etched for 9 and 12 min show higher capacitance and 1/$R_{HF}$.

The mid-frequency and low-frequency semicircles are associated with interfacial processes, including double-layer capacitance and charge-transfer resistance [57,58]. The corresponding capacitance $C_{MF}$ and resistance ${1/R}_{MF}$ from the MF RC equivalent circuits are shown in Figure 10e,f. The capacitances fall within the range of 10 nF to 10 μF, consistent with typical double-layer capacitor behavior; $1/R_{MF}$ is in the order of 10^−4^ to 4.6 × 10^−3^ Ω^−1^, and again, 1/$R_{MF}$ shows a maximum for 9 min etching. The $C_{MF}$ varies significantly between pre- and post-CV, unlike the HF parameters, as expected, and does not show any clear trend. This tendency is even more pronounced for the LF parameters, where most cells did not show a LF semicircle after the CV, at both RT and 40 °C. $C_{LF}$ (Figure 10h) is 2 to 3 orders of magnitude higher than $C_{MF}$. Similarly, $C_{HF}$ is 2 to 3 orders of magnitude higher than $C_{MF}$. All $\frac{1}{R}\propto \frac{1}{\rho }\;=\;\sigma ,$ where σ is the ionic conductivity of Na^+^ in the electrolyte: 1/$R_{HF}$, 1/$R_{MF},$ and 1/$R_{LF}$ are similar, with an optimal value for the samples etched for 9 min. Curiously the $R_{series}$ is maximum for the samples etched for 9 min, similar to the resistivity previously shown for the current collectors (Figure 8a,b). This indicates that this etching is beneficial for the mobility of the ions in the bulk electrolyte (Figure 10c,e,g) within the pouch cell and detrimental to the mobility of electrons in the Cu current collectors (Figure 8c,d and Figure 10b).

**Figure A1:**
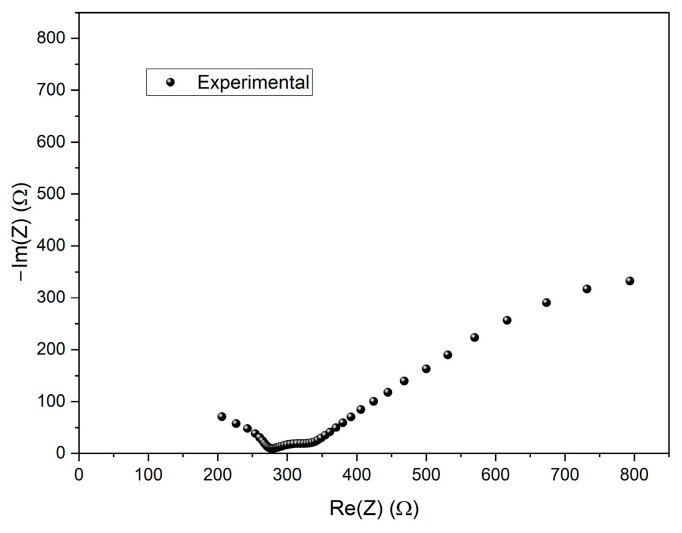
PEIS for cycle 3 for the Zn/Na_2.99_Ba_0.005_OCl/Cu-sheet (⌀0.125 mm) pouch cell assembled in batch 1.

**Figure A2:**
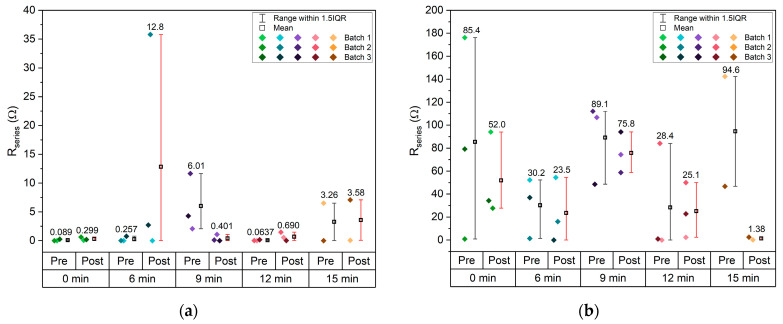
Resistance in series obtained from fit of PEIS performed at (**a**) RT (**b**) 40 °C. Values on top of dispersion bars represent the mean value.

**Figure A3:**
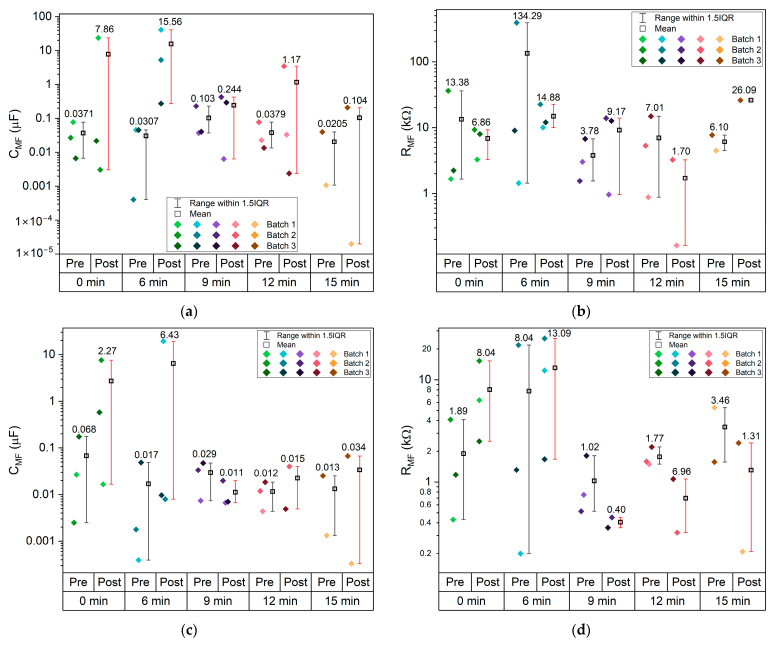
Fit parameters obtained for PEIS: (**a**) mid frequency capacitance at RT; (**b**) mid frequency resistance at RT; (**c**) mid frequency capacitance at 40 °C; (**d**) mid frequency resistance at 40 °C. Values presented in logarithmic scale for better visualization. Some data points were excluded due to unreasonably low resistance or capacitance values, which indicate that the MF semicircle was not present.

**Figure A4:**
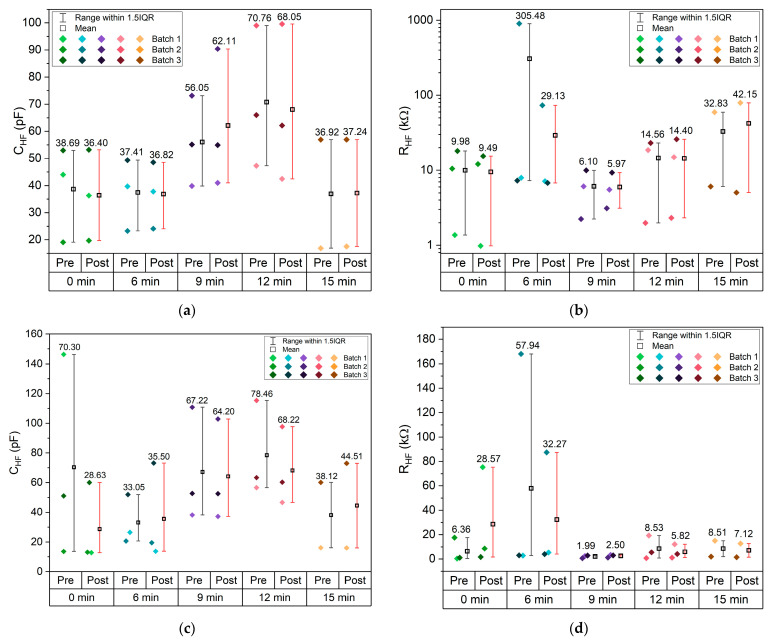
Fit parameters obtained for PEIS: (**a**) High frequency capacitance at RT (**b**) Log scale High frequency resistance at RT (**c**) High frequency capacitance at 40 °C (**d**) High frequency resistance at 40 °C. Inset plots highlight zones with lower capacitance/resistance for better visualization.

Overall, the use of thin films significantly affects the battery’s performance. Figure A1 shows the PEIS for a Zn/Na_2.99_Ba_0.005_OCl/Cu cell that used a 0.125 mm copper sheet as the positive current collector. This cell exhibited impedances in the same order of magnitude as those using copper thin films (Appendix A, Figure A1).

#### 2.2.2. Cyclic Voltammetry

**Figure 11:**
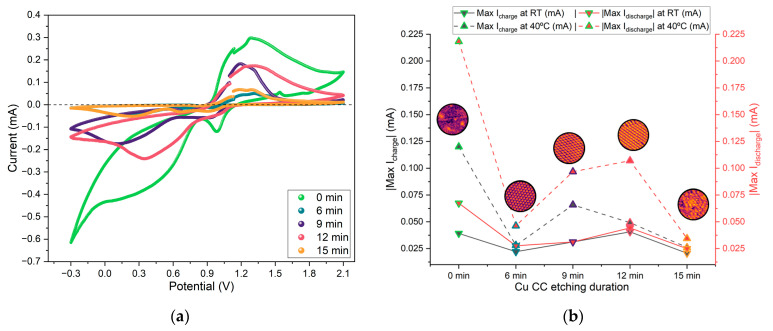
(**a**) Selected CV curves for Zn/Na_2.99_Ba_0.005_OCl/microstructured Cu cells with each current collector type; (**b**) comparison across etching duration of maximum discharge current (red) and maximum charging current (black) at RT and 40 °C. Note: effective surface area = 6.25 cm^2^.

From the CV, the peak charging and discharging currents for each cycle at RT and 40 °C were extracted and are displayed in Figure 11. This parameter reflects expected battery performance, with higher absolute values corresponding to better cell performance.

**Figure A5:**
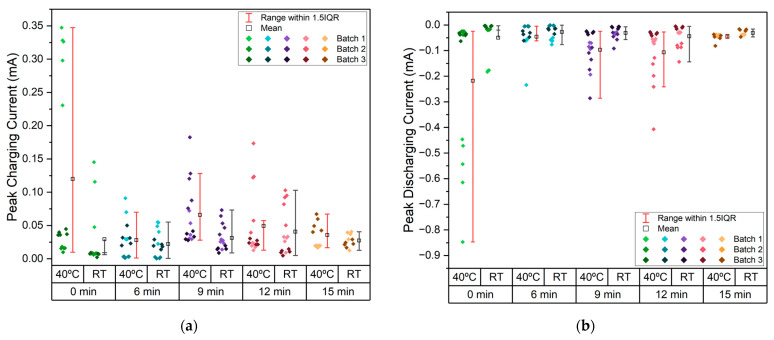
Currents at: (**a**) anodic (charging) peak and (**b**) cathodic (discharging) peak function of collector type and temperature.

**Figure A6:**
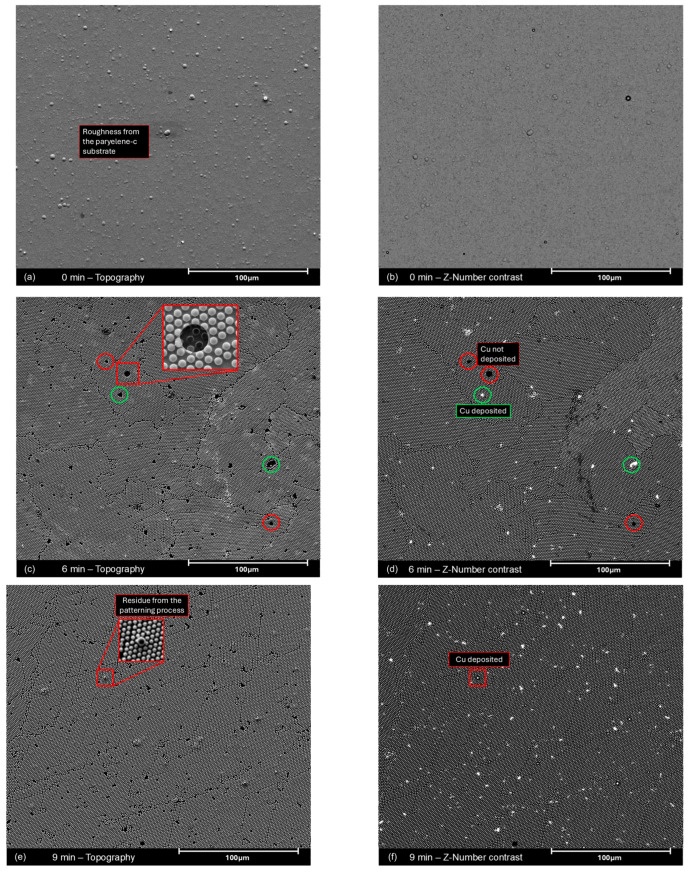

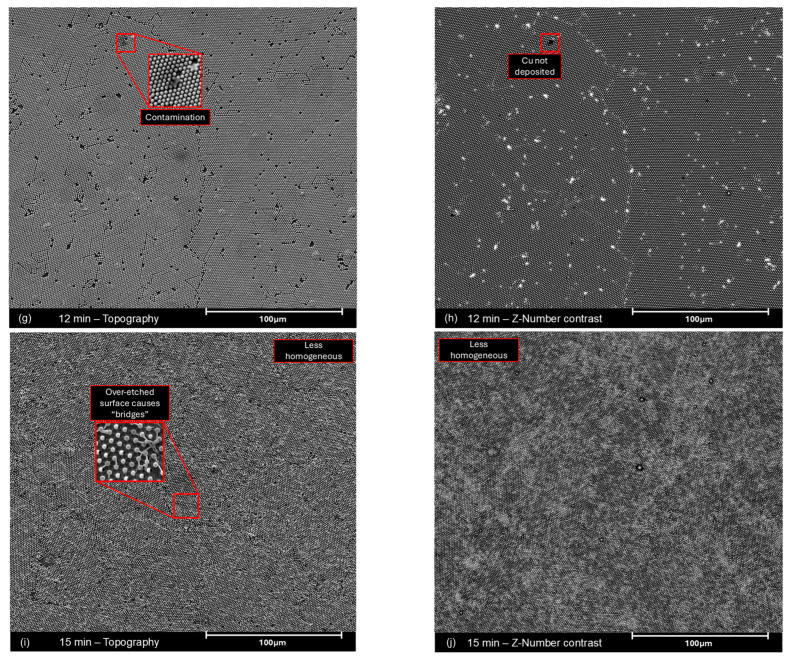
Scanning electron microscope images of the microstructured current collectors for each etching duration. (**a**,**b**) 0 min, (**c**,**d**) 6 min, (**e**,**f**) 9 min, (**g**,**h**) 12 min, (**i**,**j**) 15 min. Left side column shows images obtained with secondary electrons (topography sensitive), right side columns show images obtained with backscattered electrons (atomic number sensitive).

**Figure A7:**
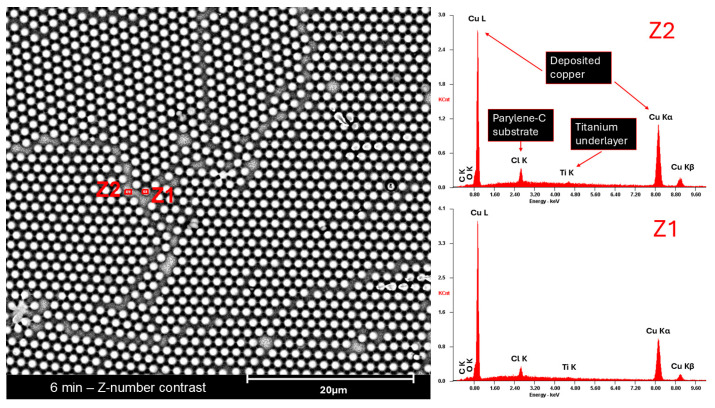
Scanning electron microscope image of the microstructured copper sample etched for 6 min. Z1 and Z2 identify the spots for Energy-Dispersive X-ray Spectroscopy analysis. On the right side are the obtained EDS spectra for each zone.

**Figure A8:**
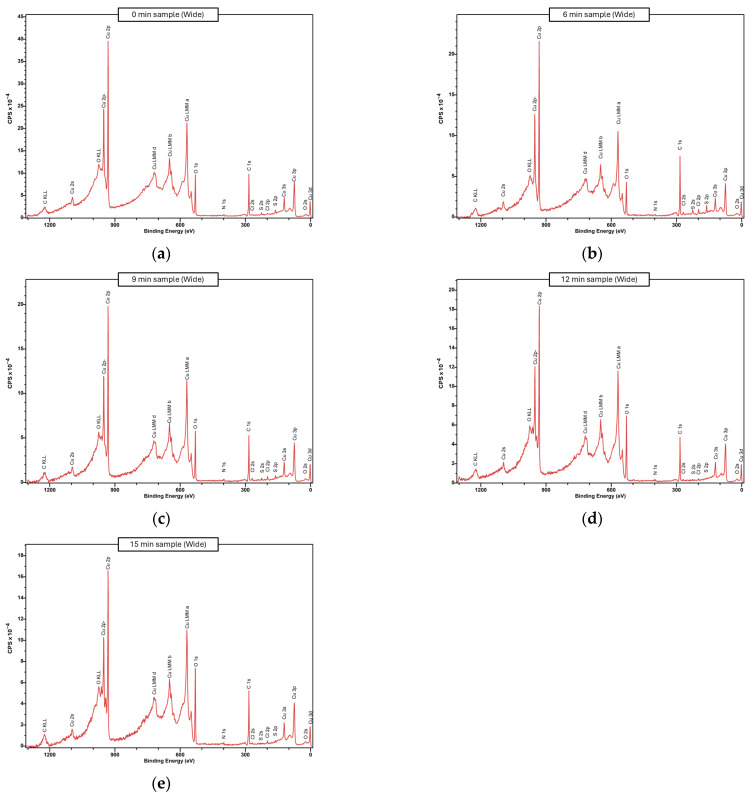
Wide XPS spectra of the microstructured copper samples for each etching duration. (**a**) 0 min, (**b**) 6 min, (**c**) 9 min, (**d**) 12 min, (**e**) 15 min.

**Figure A9:**
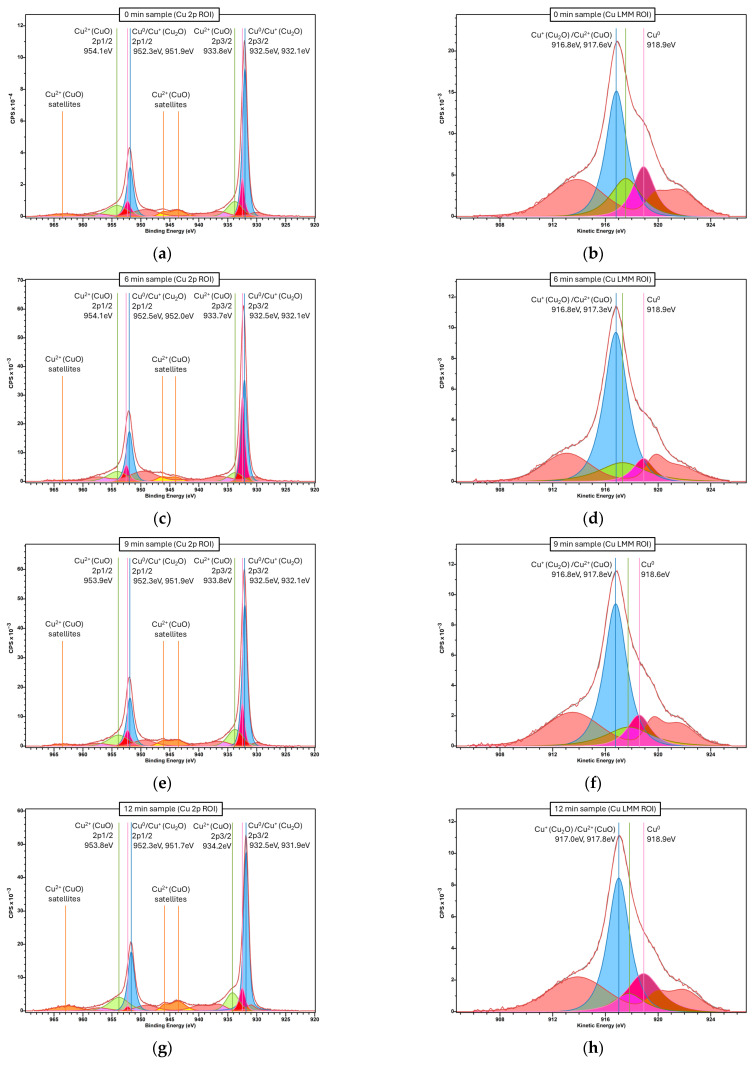

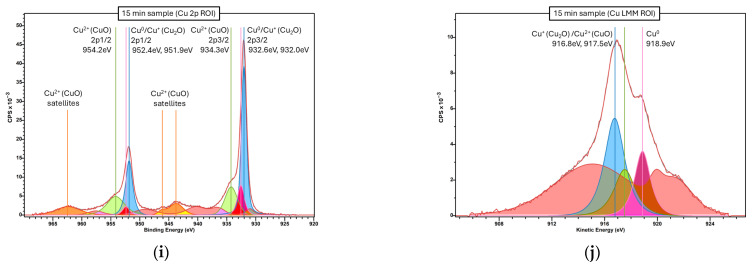
Localized XPS spectra of the microstructured copper samples for each etching duration. (**a**,**b**) 0 min, (**c**,**d**) 6 min, (**e**,**f**) 9 min, (**g**,**h**) 12 min, (**i**,**j**) 15 min. Cu 2p ROI spectra, used to distinguish Cu^+^/Cu^0^ from Cu^2+^, on the left side column. Cu LMM (L_3_M_4_,_5_M_4_,_5_) Auger ROI spectra, used to distinguish Cu^+^ from Cu^0^, on the right side column. Vertical lines and adjacent labels indicate the location of identified Cu peaks, labeled according to NIST X-ray Photoelectron Spectroscopy Database (reference [63]). Cu^+^ peaks colored in blue, Cu^0^ peaks colored in rose, Cu^2+^ peaks colored in green and loss features colored in red.

The CV data shows peak charging currents of 0.35 mA and discharging currents of 0.85 mA (Figure A5). Similar to the trend observed for the PEIS, samples etched for 9 and 12 min show slightly higher peak currents, particularly at 40 °C.

#### 2.2.3. Charge/Discharge Cycles and GITT Stability Tests

**Figure 12:**
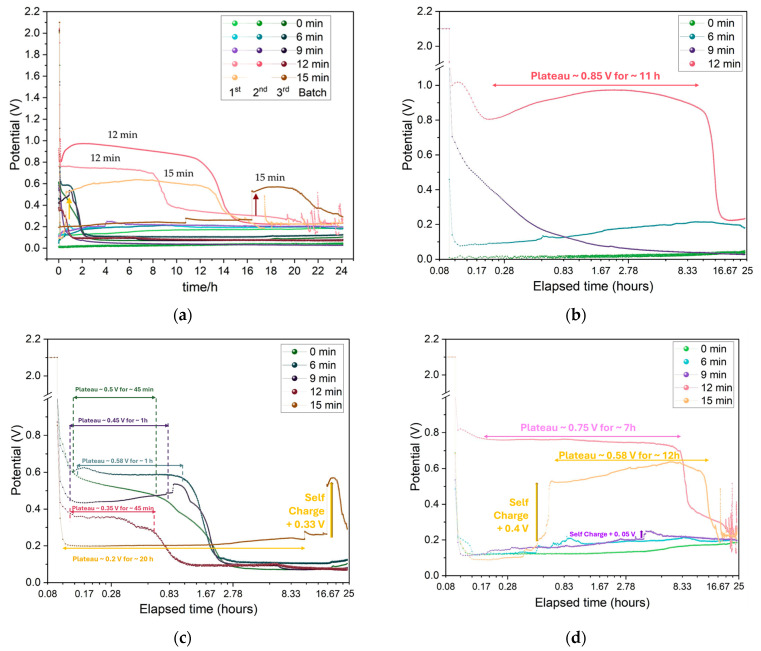
Charge/discharge cycle plots of all Zn/Na_2.99_Ba_0.005_OCl/microstructured Cu cells: (**a**) Comparison between all batches and etching times. Discharge was performed through a 26.4 kΩ or 21.7 kΩ external material resistor. (**b**) Cells from batch 1; (**c**) cells from batch 2; (**d**) cells from batch 3.

The effect shown previously for the CV maximum charge and discharge currents is also observed when the Zn/Na_2.99_Ba_0.005_OCl/microstructured Cu cells for each collector type are set to charge for three minutes (180 s) and discharge for 24 h (86,400 s) with a 26.4 kΩ or 21.7 kΩ external material resistor (Figure 12). The effective surface area of the cells is 6.25 cm^2^. The maximum discharge current was 0.038 mA, corresponding to a maximum discharge capacity of 0.56 mAh for the 24 h obtained with the samples etched for 12 min. It is noteworthy that these cells have no capacity, as there are only current collectors. The mobile ions that plate on the copper collector upon discharge have their origin in the electrolyte. The electrolyte is the only source of capacity, and its maximum capacity is ≈660 mAh.g^−1^. However, as the ions plate on both interfaces while cycling, it is not expected that the maximum capacity of the electrolyte will be reached. The cycling voltage plateau and capacities for the three batches of cells with different etch times are in agreement with the $C_{LF}$, Max I_discharge_, and CCC_bulk_ (for the balance of holes): two features of the cells and one of the individual copper current collectors related to the available number of charge carriers (herein Cu^2+^/Cu^+^). The two 15 min cells show self-charge, with arrows pointing up. This is a feature commonly observed with ferroionics of the family A_3−2x_M_x_OCl (A = Li, Na, K; and M = Mg, Ca, Sr, Ba) [59,60].

**Figure 13:**
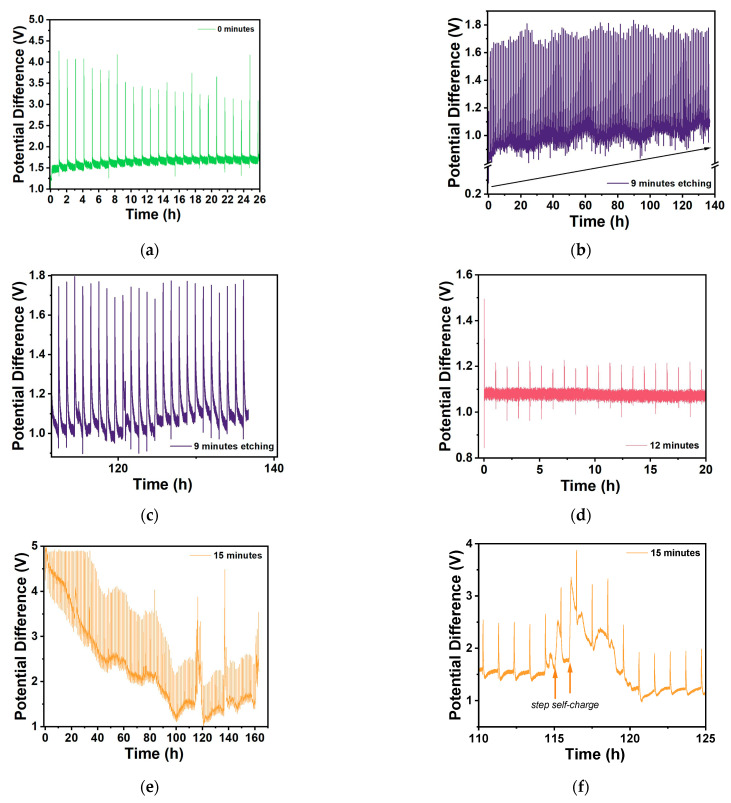
GITT performance of selected Zn/Na_2.99_Ba_0.005_OCl/microstructured Cu cells with different etching durations: (**a**) 0 min; (**b**) 9 min (140 h); (**c**) zoom between 110 h and 140 h; (**d**) 12 min; (**e**) 15 min; and (**f**) zoom of 15 min between 110 and 125 h.

**Figure A10:**
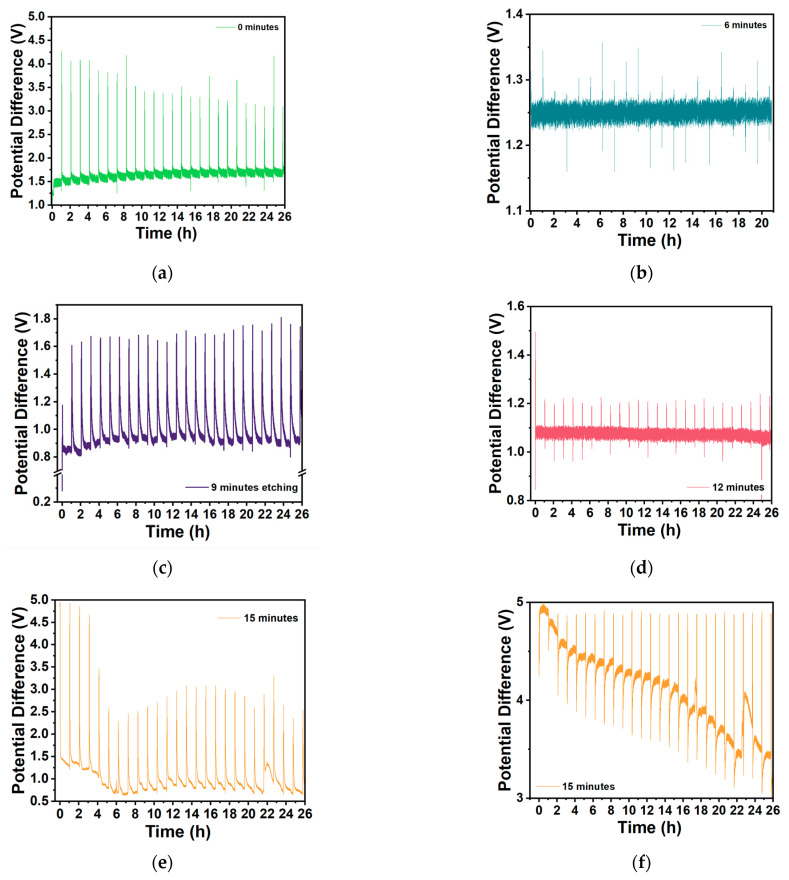
GITT performance of selected Zn/Na_2.99_Ba_0.005_OCl/Microstructured-Cu cells with different etching durations for the first 20/26 h. (**a**) 0 min, (**b**) 6 min (charging current 50 μA), (**c**) 9 min, (**d**) 12 min, (**e**) 15 min batch 3, (**f**) 15 min batch 1.

The GITT data (Figure 13and Figure A10) indicate improved OCV after cycling, demonstrating that these cells can maintain stable performance over several days, particularly those with 9 min to 15 min etching. The reduced charging current of 1 µA and time of 100 s were intentionally left unaltered to allow direct comparison between all of the cells.

The less response upon charging with a small current, the better, as it shows that the sample is stable and will support higher charge and discharge currents.

Conversely, these cells polarize immediately under the current collectors’ potential bias due to the ferroelectric electrolyte, which may indicate that the cells do not support extra charge. We would have to discharge them for many hours or use a higher discharge current before proceeding with the GITT analyses to allow the cells to charge more. However, we wanted to show the response for all of the cells with similar electrochemical “history”. The 0 min cell charges to about 4 V, which denotes that it does not hold more charge and is more unstable, showing an OCV of ~1.5 V. The 9 min cell charges to a maximum of 1.8 V, with a clear charging trend as well as self-cycling, as shown previously by the present family of electrolytes studied by the authors. The 12 min cell shows itself to be very stable when charging between 1.1 and 1.2 V, which is approximately the regular OCV of these cells. The 15 min cell starts by charging immediately to ~5 V, which is in accordance with the immediate discharge shown in Figure 12 and Figure 13e; however, it becomes more stable, with a lower potential difference amplitude after 100 h, when it also starts to self-charge, with self-charging steps at approximately 114, 136, and 159 h. After turning to OCV, the 15 min cell shows a self-charge trend during ~1 h OCV, unlike the 0–12 min samples.

#### 2.2.4. Comparison of Correlated Parameters

Although in most cases the relationships between etching duration and measured cell parameters must be taken carefully, similar trends observed between current collector parameters and cell parameters across etching times can help identify the factors underlying the cells’ behavior.

Figure 10c shows that the electrolyte conductivity (represented as 1/R_HF_) reaches its highest value after 9 min of etching. When the etching time is extended to 12 min, a sharp decrease in conductivity is observed. Based on these results, the 9 min etching condition would be expected to produce a higher maximum current in cyclic voltammetry (CV) and lead to better overall electrochemical performance during cycling. However, this trend is not confirmed by the data in Figure 11 and Figure 12. In Figure 11, the highest current is observed for the unetched (0 min) collector, even though its electrochemical cycling performance was the poorest. The next highest CV currents occurred for the 9 min and 12 min etched samples. Interestingly, when analyzing the charge–discharge cycling in Figure 12, the best performance is achieved with the 12 min etched cell. The enhanced performance of the 12 min compared to the 9 min sample collector can be attributed to the increased surface area (as shown in Figure 2b), which provides more nucleation sites for sodium metal deposition on the current collector upon cycling, improving the battery performance.

**Figure 14:**
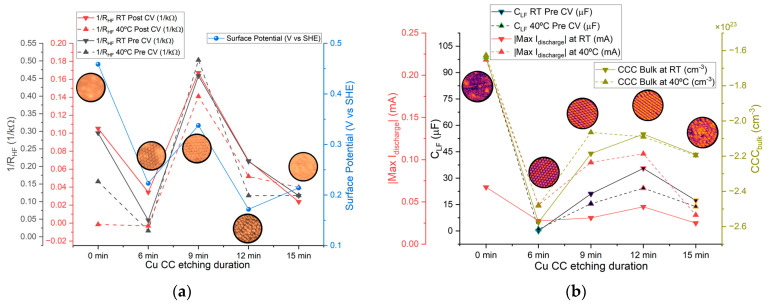
Comparison across etching durations of selected parameters (average values) for the Zn/Na_2.99_Ba_0.005_OCl/microstructured Cu cells (left axis) and microstructured Cu current collector parameters (right axis): (**a**) ${1/R}_{HF}$ at RT and 40 °C and surface potential vs. etching time; (**b**) pre-CV $C_{LF}$ at RT and 40 °C, Max I_discharge_ at RT and 40 °C, and CCC_bulk_ (electrons) at RT and 40 °C.

Figure 14a shows a comparison between the average ${1/R}_{HF}$ and surface potential across all etching durations. Both parameters exhibit similar trends, indicating that higher surface potential generally corresponds to higher ${1/R}_{HF}\propto \sigma_{{Na}^{+}}$; in other words, the difference in chemical potentials between the Zn (−0.76 V, SHE) and Cu current collectors (+0.34 V, SHE for 9-min) $∆V\;=\;-\mu \left(Zn,SHE\right)+\mu \left(Cu,SHE\right)\;=\;1.10$ V controls the driving force (electric field inside the cell) that influences the ionic conductivity. As previously discussed, the force acting on charge carriers within the cell is primarily determined by the difference in chemical potential between the opposing current collectors/electrodes. A lower surface potential of the copper current collector (higher in the SHE scale used herein) increases its potential difference relative to the zinc current collector, effectively enhancing charge carrier mobility (Na^+^) across the cell and reducing the cell’s bulk resistance.

Figure 14b presents the average values of $C_{LF}$, Max I_discharge_, and CCC_bulk_ (for electrons). In this case, increased $C_{LF}$ correlates with higher discharge currents. A similar trend is observed for CCC_bulk_ (for holes or Cu^2+^ and Cu^+^): lower charge carrier concentrations of electrons correspond to higher $C_{LF}$ and Max I_discharge_. This suggests that the Cu/electrolyte interface influences performance through changes in capacitance, which are linked to charge concentrations at the interfaces. All of the latter are correlated with the dynamic tendency to align the Fermi levels (electrochemical potentials $\bar{\mu }$) between the electrolyte and the Cu current collector/positive electrode.

**Figure 15:**
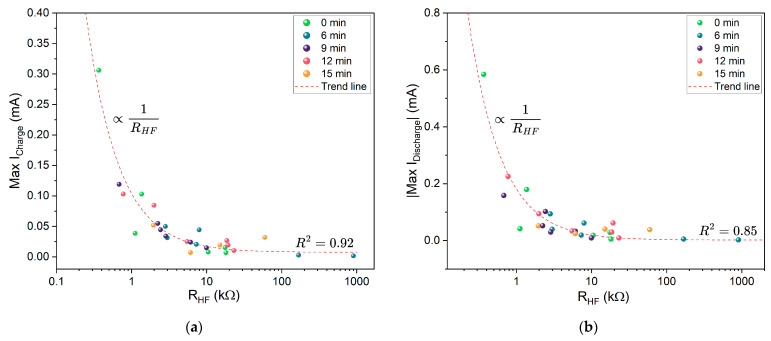
Correlation between maximum cyclic voltammetry (CV) currents and $R_{HF}$: (**a**) Maximum charging current vs. $R_{HF}$. (**b**) Maximum discharging current vs. $R_{HF}$. The red dashed line indicates the ${1/R}_{HF}$ trend line; R^2^ denotes the corresponding coefficient of determination.

The bulk resistance of the cell generally makes up a significant portion of the total resistance. The relationship between bulk resistance ($R_{HF}$) and maximum charge and discharge currents is shown in Figure 15. Overall, lower bulk resistance correlates with higher peak currents. This correlation highlights the importance of enhancing charge mobility within the electrolyte by reducing overall resistance, either by increasing the potential difference across the current collectors or by adjusting the electrolyte composition and morphology.

Taken together, these observations highlight the interconnected role of the current collector surface potential, charge carrier concentration, and capacitance in governing cell performance. While etching time alone does not directly determine cell behavior, its influence on current collector properties indirectly shapes critical parameters such as resistance and maximum discharge current. Understanding how modifications at the Cu/electrolyte interface alter charge transport mechanisms offers a practical pathway to optimizing cell design and improving overall efficiency.

## 3. Materials and Methods

### 3.1. Fabrication of the Microstructured Copper Current Collectors

**Figure 16:**
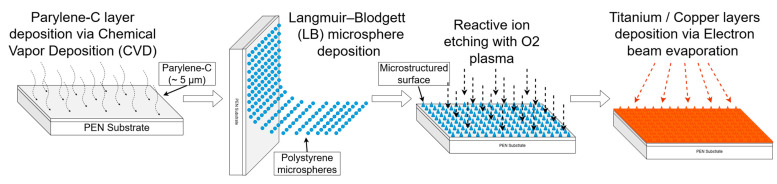
Diagram of the four-step fabrication process for the microstructured copper current collectors.

The fabrication of microstructured copper current collectors was based on a bottom–up templating approach combining parylene-C coating, colloidal microsphere assembly, reactive ion etching, and electron-beam evaporation (Figure 16).

#### 3.1.1. Parylene-C Deposition

As a base layer, parylene-C was deposited onto polyethylene naphthalate (PEN) substrates, which served as flexible supports for subsequent microstructuring. Prior to deposition, the PEN substrates were cleaned with isopropanol followed by deionized water. Parylene-C deposition was performed via chemical vapor deposition (CVD) using a PDS-2010 Labcoater 2 system (Specialty Coating Systems), following procedures described in Ref. [61].

The final film thickness was controlled by the mass of the parylene-C dimer precursor and verified using a profilometer. A dimer mass of ~8 g yielded films with thicknesses ranging from 4 to 5 μm. The parylene-C precursor (chloro-para-xylylene dimer) was sourced from SCS (Specialty Coating Systems).

#### 3.1.2. Microsphere Deposition

To create a self-assembled etching mask, polystyrene microspheres were deposited onto the parylene-coated substrates via the Langmuir–Blodgett (LB) technique, as described in Ref. [62]. A colloidal suspension was prepared by diluting a 10% microsphere solution (1.30 ± 0.03 μm diameter, Microparticles GmbH, Berlin, Germany) at a 1:1 volume ratio with a diacetone-based solution containing 1% styrene and 0.1% sulfuric acid.

Before deposition, the LB trough (Biolin Scientific KSV NIMA, Gothenburg, Sweeden) was cleaned with deionized water, followed by ethanol. The tank was then overfilled with 700 mL of deionized water to stabilize the air–liquid interface. A total of 400 μL of the colloidal solution was gently added to the interface using a microsyringe. The system’s barriers were closed at 5 mm/min, facilitating self-assembly of the microspheres into a close-packed hexagonal monolayer. This monolayer was then transferred onto the parylene-coated substrate by vertically withdrawing the substrate from the water surface at the same speed (5 mm/min).

#### 3.1.3. Reactive Ion Etching (RIE)

To modify the geometry of the polystyrene spheres and, thus, tailor the resulting surface microstructure, oxygen plasma etching was conducted using a Trion Phantom 3 RIE system. The etching conditions were fixed at 50 W power, 20 mTorr O_2_ pressure, and 100 sccm gas flow, with the etch durations varied at 0, 6, 9, 12, and 15 min to systematically control the final feature morphology. After RIE, the remaining microspheres were removed by sequential ultrasonic cleaning in toluene and isopropanol.

#### 3.1.4. Copper Deposition

Final metallization was performed by electron-beam evaporation in a custom-built vacuum system. Prior to deposition, the chamber was evacuated to a base pressure of (1–2) × 10^−6^ mbar. A 20 nm titanium adhesion layer was first deposited, followed by 250 nm of copper. Both metals were deposited using separate crucibles containing high-purity pellets. Film thickness was monitored in real time using a quartz crystal microbalance.

This sequence yielded microstructured Cu current collectors with tunable surface morphology, suitable for interface engineering in solid-state battery configurations.

### 3.2. Characterization of the Microstructured Copper Current Collectors

#### 3.2.1. Charge Transport Measurements

The sheet resistivity of the copper current collector samples was measured using the van der Pauw method, employing a Linseis HCS 1 Hall-effect measurement system. Each sample was prepared with four collinear electrical contacts placed along the edges. A constant current of 3 mA was applied between two adjacent contacts, and the resulting voltage was measured between the remaining two contacts. This configuration allowed for the calculation of resistivity using standard van der Pauw equations. Charge carrier density and mobility were measured using the same setup and equipment, while applying a 3 mA current for the Hall-effect measurement. Measurements were performed as a function of temperature, from 25 °C up to 70 °C, in controlled inert conditions. Each temperature was equilibrated. The chamber was initially evacuated to 10^−2^ bar and then purged continuously with high-purity nitrogen at 4–5 L/min. This minimized spurious surface reactions, stabilized the thermal conditions, and reduced electrical noise during measurements.

#### 3.2.2. Topography and Surface Potential Measurements

Atomic Force Microscopy (AFM) was used to perform topographical and surface potential analyses of the copper current collectors using an MFP-3D Infinity AFM (Asylum Research). A minimum of eight regions were imaged per sample (2 × 2 cm), using Ti/Ir-coated silicon tips (radius 25 nm ± 10 nm). Images were captured in tapping mode over 20 × 20 μm^2^ areas (256 × 256 pixels). Scan rates were adjusted individually to minimize imaging artifacts such as parachuting.

To enhance visual clarity for publication, raw 256 × 256 topography maps obtained from AFM were upscaled to 1024 × 1024 pixels using cubic interpolation (skimage.transform.resize, order = 3) with anti-aliasing enabled. This processing was carried out solely for visualization purposes; all quantitative analysis was performed on the original-resolution data. Color scaling, scale bars, and labels were applied programmatically to maintain consistency across images. The processed images were saved as high-resolution PNG files (300 dpi) using custom Python scripts written in Jupyter Notebooks.

For each AFM image (minimum of eight per sample type), various quantitative parameters were extracted to statistically characterize the surface roughness, structure density, and spatial uniformity:

1. Root-Mean-Square Roughness (RMS): Automatically calculated using Igor Pro 6 (Asylum Research). RMS quantifies the average deviation in height across the scanned area and serves as a standard metric for surface roughness. A higher RMS value indicates a rougher and more topographically varied surface.
2. Surface Area Increase (%): Also obtained via Igor Pro. This parameter reflects the percentage increase in total surface area compared to an ideal flat 2D surface (20 × 20 μm = 400 μm^2^). It captures the increased electrochemically active area made available by the 3D structuring.
3. Number of Particles: Computed via Python 3.11.9 using local maxima detection (skimage.morphology.h_maxima) with a height prominence threshold of 20 nm to isolate individual topographical features (cone-like structures). This metric provides insight into structural density and inter-sample or intra-sample consistency. A uniform particle count across zones indicates good fabrication repeatability.
4. Average Distance to Six Nearest Neighbors (D_6NN_): Using the particle centroids identified above, the average distance to each particle’s six closest neighbors was calculated. The particles tend to form hexagonal arrangements, and this metric captures the characteristic spacing between them. This is relevant to understanding how the electrolyte penetrates between structures, as well as where sodium deposition may preferentially occur.
5. Standard Deviation of Nearest-Neighbor Distance (σ_DNN_): For each particle, the distance to its single closest neighbor was computed, and the standard deviation of these distances across each image was used to quantify local ordering. Lower values of σ_DNN_ indicate high spatial uniformity (ordered structure), while higher values suggest disorder or irregular spacing. These parameters were computed individually for each AFM frame, allowing for statistical comparison across etching conditions using box plots.
6. All non-native analyses were conducted using custom Python scripts in Jupyter Notebooks.

Surface potential measurements were obtained via KPFM using the same scan parameters as AFM (20 × 20 μm, 256 × 256 pixels). Due to occasional tip–sample disconnection events—observable as abrupt potential drops—data points with values below 0 V vs. SHE were masked and excluded from the quantitative analysis. These masked zones typically represent areas where negative charge accumulated and where there was subsequently a breakdown. To ensure accurate surface potential statistics (mean and standard deviation), a custom Python script applied a threshold-based mask (V_z_ < 0 V) to the raw data before calculating these parameters. For visualization, masked regions were overlaid with a semi-transparent dark blue layer, and the upscaled (1024 × 1024) voltage maps were saved as high-resolution PNGs. Color bars were dynamically scaled to valid data ranges, with appropriate unit labels (V or mV).

Quantitative surface potential analysis (SKPM) followed the masking for surface potential < 0 V from each SKPM image. Using custom Python code, the average surface voltage was calculated as the arithmetic mean of all unmasked pixel values. This parameter represents the average local surface potential of the sample relative to the conductive AFM tip and provides insight into the average electronic environment at the sample surface. These parameters were calculated for each SKPM image individually. With multiple regions analyzed per sample, statistical comparisons between etching conditions were carried out using box plots to capture variability within and across samples. Dual-pass KPFM is used to minimize crosstalk from topography effects. The first pass acquires topography in tapping mode, and the second pass rescans the conducting tip at a constant relative height above the surface.

#### 3.2.3. Scanning Electron Microscopy and X-Ray Photoelectron Spectroscopy

An FEI QUANTA 400 FEG ESEM scanning electron microscope was used. The chemical composition analysis was performed with an EDAX Genesis X4M energy-dispersive X-ray spectrometer. Magnifications of 1000× and 25,000× were used with an energy of 15 keV in both backscattered electron detection and secondary electron detection. To perform the X-ray Photoelectron Spectroscopy analysis, a Kratos Axis Ultra HSA system was employed. The primary goal was to detect the presence of oxidized copper (Cu^2+^, Cu^+^, Cu^0^) and residue from the fabrication process.

### 3.3. Separator Preparation and Cell Assembly

The electrolyte used in this study was Na_2.99_Ba_0.005_OCl, a glassy ferroelectric solid-state conductor synthesized via aqueous solvation, following the procedure described by Braga et al. [26,31]. The synthesis utilized NaCl (99.0%), NaOH (anhydrous, 99%), and Ba(OH)_2_ (anhydrous, 94–98%) as precursor materials.

Following synthesis and thorough drying, the solid electrolyte powder was mechanically processed using a FRITSCH Planetary Mono Mill PULVERISETTE 6. Milling was performed in a sealed agate vessel containing five agate balls (20 mm diameter), operated at 300 rpm for 40 min to achieve uniform particle size reduction under controlled inert conditions.

The milled electrolyte was subsequently blended with the polyvinyl acetate (PVAc) binder with a weight ratio of 80:20 (Na_2.99_Ba_0.005_OCl:PVAc). This composite mixture was then re-milled using the same planetary ball-mill configuration to ensure homogeneity and cohesive powder formation.

To fabricate the separator membrane, the composite powder was dispersed in ethanol to form a viscous, homogeneous slurry, which was impregnated onto 3 × 3 cm cellulose sheets serving as mechanical supports. These coated sheets were subsequently placed in a vacuum oven at 40 °C and −500 mbar (relative to atmospheric pressure) for a minimum of 24 h, ensuring complete solvent removal and solidification of the membrane.

Zinc current collectors measuring 25 × 25 mm^2^ were cut from a high-purity zinc sheet (99.98%) and used as the negative metal electrode in the assembled cells.

Cell assembly was carried out under ambient conditions, outside a glovebox. The prepared separator was manually positioned between the zinc and copper current collectors, ensuring that no direct electrical contact occurred between the electrodes. Teflon tape was applied around the perimeter to mechanically secure the assembly and maintain alignment.

Finally, the cells were vacuum-sealed using a pilot-scale packaging line. An aluminum-laminated pouch was first formed using a pouch case-forming machine. Subsequently, the top and one side of the pouch were sealed using a top-and-side heat-sealing machine. The partially sealed pouch was placed into a vacuum pre-sealing chamber, where air was evacuated. The final seal was then applied to the remaining side, completing the encapsulation and ensuring environmental isolation of the cell components.

### 3.4. Pouch Cell Electrochemical Characterization

To assess the electrochemical performance of the Zn/Na_2.99_Ba_0.005_OCl/structured Cu solid-state cells, potentiostatic electrochemical impedance spectroscopy (PEIS) and cyclic voltammetry (CV) measurements were performed using a Biologic VMP-300 potentiostat. The cells were tested at both room temperature and 40 °C. The cells were heated up to 40 °C inside a thermostat-controlled sand bath, where they were maintained for at least 2 h before being tested.

#### 3.4.1. Electrochemical Impedance Spectroscopy (PEIS)

PEIS was employed to evaluate the internal resistance of the assembled cells. The measurements were conducted at open-circuit voltage (OCV) by applying a 10 mV AC perturbation across a broad frequency range from 7 MHz down to 100 mHz. The resulting spectra were analyzed by equivalent circuit fitting, using EC-Lab V11.61.

#### 3.4.2. Cyclic Voltammetry (CV)

The CV cycles were performed at a scan rate of 5 mV.s^−1^, and the experiment was repeated five times to assess cycle-to-cycle reproducibility. The voltage window for each cycle was set between −0.3 V (discharge limit) and +2.1 V (charge limit).

This testing protocol enabled identification of any irreversible processes or degradation over the initial cycles, as well as insight into how surface structuring of the current collector may influence redox kinetics and interfacial stability.

#### 3.4.3. Charge/Discharge Cycles and GITT

Charge/discharge cycles were conducted with an external material resistance (either 26.4 kΩ or 21.7 kΩ) connected in parallel to each cell. The charging voltage was set to 2.1V. The GITT measurements were carried out using a Neware CT-4008Tn-5V-20mA battery tester (Montclair, CA, USA). Each test cycle consisted of charging the cell at 1 µA for 100 s, followed by a 1 h rest period.

### 3.5. Usage of Generative Artificial Intelligence

GenAI was used in the generation and debugging of the Python code used for data analysis. It was also used to improve text fluency and formatting.

## 4. Conclusions

In this study, we investigated the influence of microstructured copper current collectors on the electrochemical performance of Zn/Na_2.99_Ba_0.005_ClO/Cu solid-state batteries.

By employing a bottom–up colloidal lithography approach followed by reactive ion etching, we systematically varied the surface topography of copper films deposited on flexible parylene-C-coated PEN substrates.

Surface characterization revealed that etching times of up to 15 min led to an increase in surface area of approximately 30%, accompanied by progressively rougher and irregular topographies. KPFM measurements showed average surface chemical potential values between 189 mV and 459 mV, comparable to clean or mildly oxidized copper. The microstructured samples had lower surface potential, likely resulting from mixed effects of oxidation and topography. However, the variation in the surface potential matches the variation in the inverse of the bulk resistance in the pouch related to the ionic conductivity in the bulk electrolyte.

Charge transport characterization demonstrated that the resistivity of the thin-film copper layers remained within the range of ~1.96 to 4.5 µΩ.cm, close to the values of bulk copper. On the other hand, charge carrier density and mobility seemed to be affected by the microstructuring, with structured samples showing higher charge carrier density (electrons), in the order of 10^23^ cm^−3^, but lower electron mobility, between 6 and 18 cm^2^.V^−1^.s^−1^. We highlight that a higher concentration of electrons corresponds to lower $C_{LF}$, Max I_discharge_, and CCC_bulk_ (holes or Cu^2+^, Cu^+^).

Electrochemical characterization of the assembled Zn/Na_2.99_Ba_0.005_OCl/microstructured Cu pouch cells showed that, of all of the cells, the ones showing higher average deviation of nearest neighbors (σ_DNN_) were the best performing (12 and 15 min). However, SEM analyses actually showed that the samples showing the lowest absolute σ_DNN_ (9 and 12 min) seemed to show fewer grain boundaries and spurious defects. The 15 min sample appears very ordered with secondary low magnification, but with backscattered electrons it shows two slightly different phases enabled by “bridges” that form with etching. These cells, showing average surface potential of +0.214 V vs. SHE, also show similar relative concentrations of CuO (Cu^2+^)/Cu_2_O (Cu^+^)/Cu^0^.

Overall, the electrochemical characterization revealed a strong connection with the electrochemical characteristics of the Cu current collectors. Higher surface potential of the copper current collector correlates with increased Na^+^ ionic conductivity, as shown by the parallel trends between surface potential and 1/R_HF_. This indicates that the potential difference between the Zn and Cu collectors, governed by their chemical potential, directly affects the driving force for ion transport and helps reduce the cell’s bulk resistance. Additionally, higher low-frequency capacitance was correlated with increased discharge currents, while lower charge carrier concentrations (electrons) in the copper current collector also corresponded to higher capacitance and discharge currents. These trends indicate that the Cu/electrolyte interface affects cell performance through variations in interfacial capacitance linked to charge concentrations and the alignment of electrochemical potentials. Order (information) parameters are also likely related to the performance of the cell or, even more likely, the increased surface area at the copper current collector/electrode.

## Data Availability

Data will be made available upon request.

## Abbreviations

The following abbreviations are used in this manuscript:

PEN	Polyethylene Naphthalate
PET	Polyethylene Terephthalate
PS	Polystyrene
RIE	Reactive Ion Etching
AFM	Atomic Force Microscopy
KPFM	Kelvin Probe Force Microscopy
vdP	Van der Pauw (method)
RMS	Root Mean Square
WF	Work Function
SHE	Standard Hydrogen Electrode
RT	Room Temperature
PEIS	Potentiostatic Electrochemical Impedance Spectroscopy
EC fit	Equivalent Circuit Fitting
CV	Cyclic Voltammetry
CPE	Constant Phase Element
Ma	Modified Restricted Diffusion Element
HF/MF/LF	High Frequency/Mid-Frequency/Low Frequency
C_HF_, C_MF_, C_LF_	Capacitances Associated with HF, MF, and LF Semicircles, Respectively
R_HF_, R_MF_, R_LF_	Resistances Associated with HF, MF, and LF Semicircles, Respectively
RC circuit	Resistor–Capacitor Circuit
CVD	Chemical Vapor Deposition
LB	Langmuir–Blodgett (Technique for Depositing Monolayers)
sccm	Standard Cubic Centimeters per Minute (Unit of Gas Flow Rate)
PVAc	Polyvinyl Acetate
OCV	Open-Circuit Voltage
CC	Current Collector
CCC_bulk_	Charge Carrier Concentration of the Bulk CC
